# Scombroid and Anaphylaxis: Different Diseases Despite Featural Similarities

**DOI:** 10.7759/cureus.98497

**Published:** 2025-12-04

**Authors:** Nuno Oliveira, Vania Junqueira, Rosa Amorim

**Affiliations:** 1 Internal Medicine, Unidade Local de Saúde do Oeste, Caldas da Rainha, PRT

**Keywords:** allergology, allergy and anaphylaxis, antihistamine, generalized rash, medical dermatology, scombroid fish poisoning, scombroid syndrome

## Abstract

Food toxicity is a common cause of morbidity worldwide. Presentations of food toxicity can take diverse forms, depending on the underlying mechanisms. Scombroid is one of the most frequent forms of food toxicity; however, its exact incidence remains unknown owing to similarities with other pathologies. Scombroid is a type of food poisoning caused by the ectopic consumption of histamine that mimics angioedema. Here, we present the case of a 36-year-old male patient who presented to the emergency department (ED) after consuming tuna, with a clinical picture of generalized erythema accompanied by pruritus and gastrointestinal symptoms, without dyspnea, lip or tongue edema, and no known allergies. The patient responded well to an H1 antihistamine, with no relevant analytical changes or hemodynamic stability during observation in the ED.

## Introduction

Scombroid is a type of food toxicity, the nomenclature of which originates from the word “scombrids,” the zoological classification of the fish family encompassing tuna, mackerel, bonito, and wahoo. This syndrome is characterized by abdominal pain, diarrhea, nausea, vomiting, facial or generalized rash, urticaria and/or angioedema, tachycardia, headache, dizziness, and xerostomia [1-4]. Unlike an anaphylactic reaction, which is an IgE-mediated pathology, scombroid is caused by the ingestion of an ectopic histamine source. The clinical presentation generally occurs within 10-30 minutes of ingestion [1,2].

Scombroid is usually benign and self-limiting, whereas anaphylaxis can have fatal outcomes. Under normal conditions, histamine is not present in fish at pathological concentrations. However, if captured fish are exposed to warmer temperatures after death, histidine conversion to histamine can increase. This process is inactivated at a temperature of 0°C. Therefore, storage at temperatures of 0°C must be implemented immediately after capture. Importantly, cooking fish does not inhibit the conversion process, and fish contaminated with high histamine levels have a normal appearance and taste [1-4]. Here, we present a case of scombroid mimicking anaphylaxis.

## Case presentation

**Figure 1:**
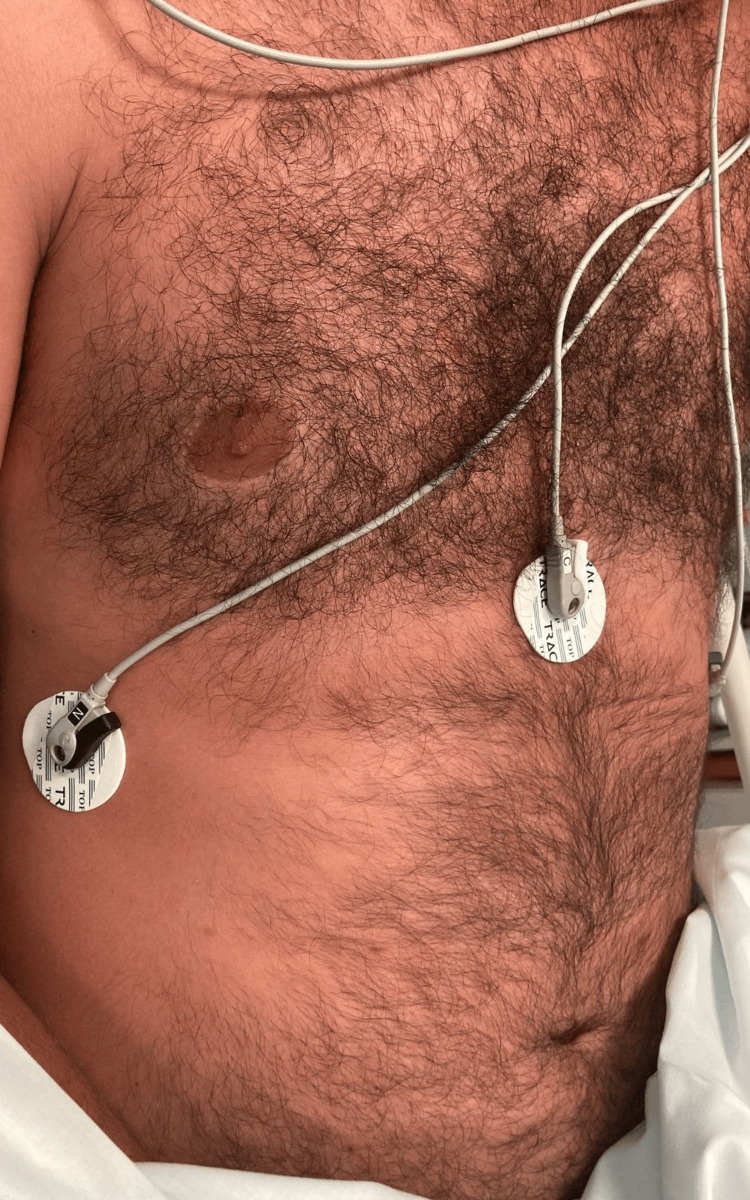
Patient’s torse showing exuberant erythema with reddish color in the torso and limbs.

**Figure 2:**
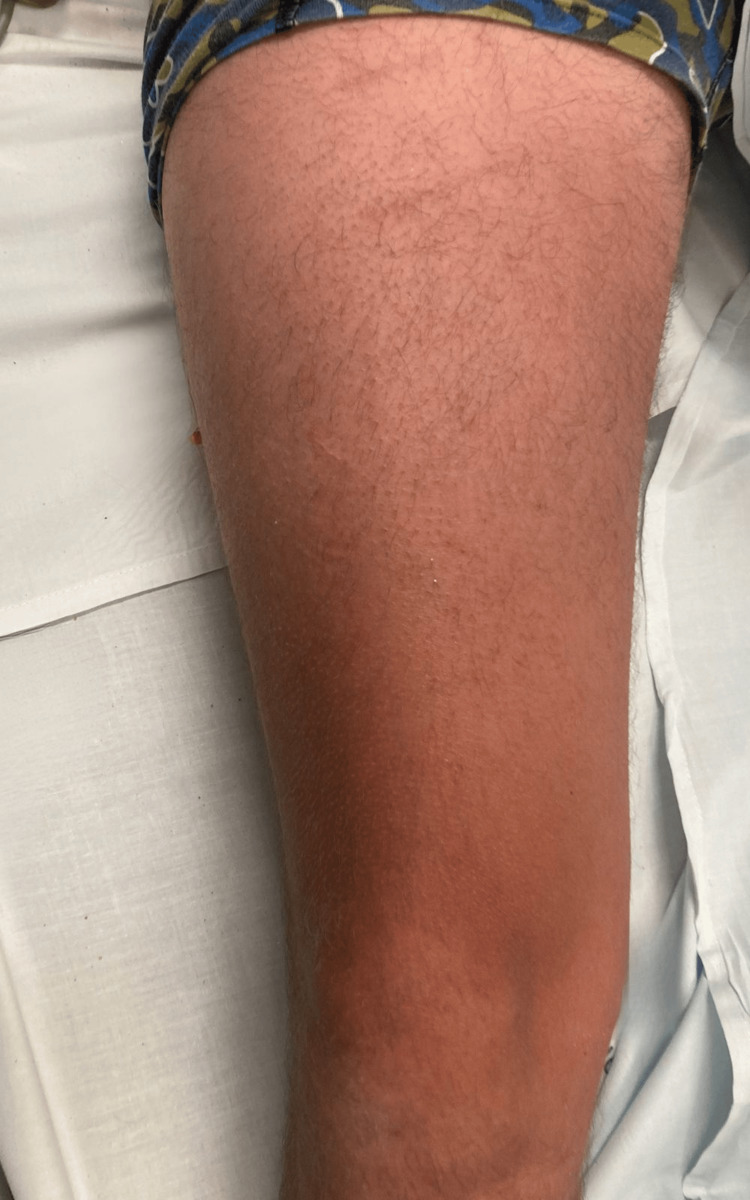
Right thigh showing dispersed erythema on both in the anterior and posterior aspect of the thigh.

**Figure 3:**
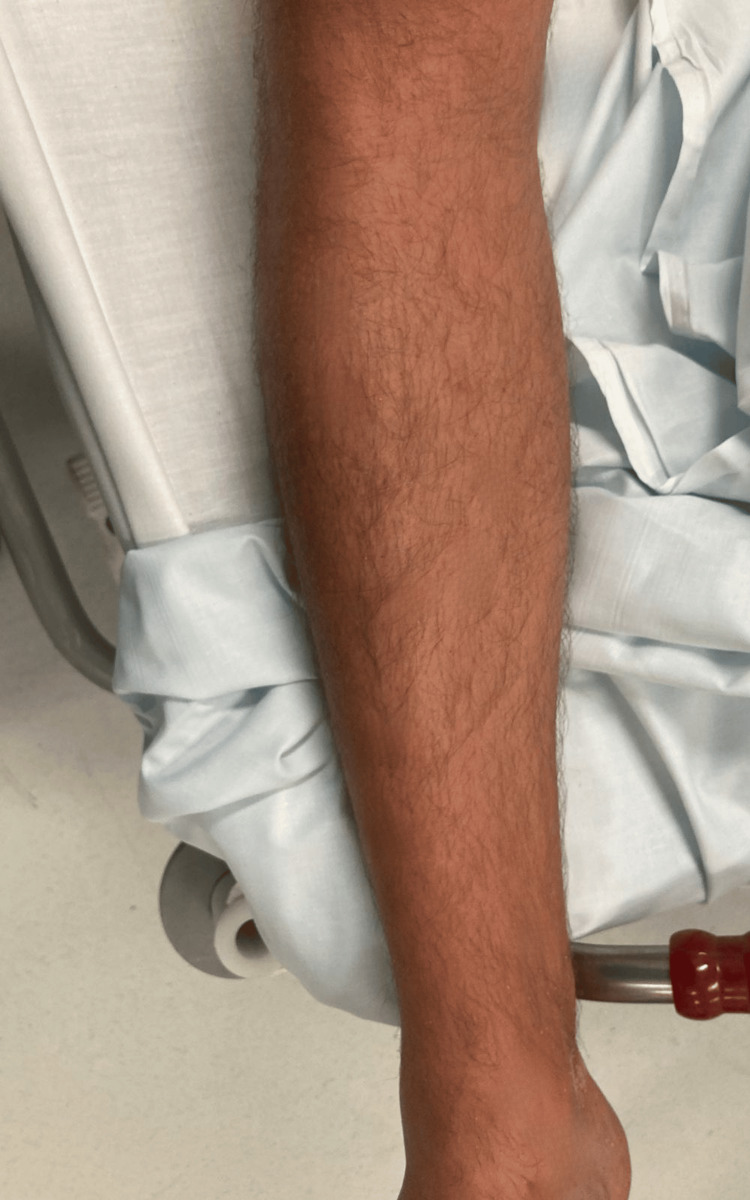
Continuity of the erythema across the leg, excluding the folding areas.

A 36-year-old Dutch man was brought to the emergency department by his mother after experiencing nausea, vomiting, and diarrhea approximately 45 minutes after a sushi meal. During triage, the nurse observed that the patient had exuberant erythema. In the emergency room, the patient presented with a blood pressure of 134/75 mmHg, heart rate of 87 beats per minute, and oxygen saturation of 95%. Hemodynamic stability was maintained throughout the patient’s stay. The patient did not present with any edema of the lips, tongue, or glottis; however, he complained of pruritus and had scattered rashes on the head, upper limbs, and torso (Figures 1-3).

The patient reported recent sushi consumption without any suspicion of the fish’s organoleptic qualities (no foul smell or flavor). An allergic condition was assumed, and hydrocortisone, clemastine, and methylprednisolone were initiated. During the first observation, his mother’s clinical complaints were unknown. Only later were we informed of the mother’s gastrointestinal symptoms, which changed the perspective of the case, as this dual presentation indicated that the consumed fish was not in the best condition. The patient showed no analytical changes, namely, his leukocyte, eosinophil, and C-reactive protein levels were normal. The patient’s airway remained stable.

Given the hemodynamic stability presented by the patient since admission and the diagnosis of scombroid, we expected good recovery with a short stay in the emergency room (ER). After the initial treatment, the erythema became less intense, and pruritus diminished considerably; however, the patient remained under surveillance for 12 hours. The patient was discharged with an H1 antihistamine medication. At discharge, the patient did not have any erythema or pruritus. Unfortunately, no post-treatment images were available.

## Discussion

The symptoms exhibited by this patient can result from excessive histamine consumption by scombroid fish; however, they are not exclusive to this etiology. Several studies have shown that pure histamine consumption does not have the same clinical effects [5,6], and the mechanism underlying their development remains unclear.

Elevations in fish tissue histamine concentrations begin after death. Although histidine is converted to histamine during the fish’s lifecycle, a peak histamine level is observed only after death, and increases are driven by an ectopic source of histidine decarboxylase from bacterial conversion in the fish gut, gills, and skin [1-5]. The most commonly associated bacteria are gram-negative commensal bacteria [5]. To prevent histamine formation and consequent scombroid poisoning, fish need to be maintained at 0°C after capture. Once the fish is contaminated, cooking results only in bacterial destruction; the accumulated histamine is not eliminated.

The most common symptoms of scombroid are rash, erythema (not on the limbs), respiratory distress, nausea, vomiting, diarrhea, epigastric pain, cramping, hypo or hypertension, and headache. The differential diagnosis of patients presenting with these symptoms should include IgE-mediated allergy and food poisoning [5-10]. In the present case, an IgE-mediated allergy was unlikely, given the patient’s clinical stability. Serum tryptase, urinary histamine metabolites, or prick tests were unavailable in the ER; hence, it is not possible to discard the hypothesis that the patient’s good presentation was not due to positive clinical evolution. Regarding food poisoning, both dermatological and gastrointestinal symptoms were noted in this patient. While diagnosis may be more challenging in some cases, this case should remind us of the importance of considering alternative diagnoses.

## Conclusions

Despite being indolent, the resemblance of scombroid to anaphylaxis makes diagnosis difficult, especially in an emergency, when there is involvement of two or more organ systems. Given the fatal consequences of anaphylaxis, clinicians are taught to treat generalized erythema as an emergency. In this context, the systematization of emergency training can momentarily suppress clinical assessment by placing unnecessary stress on the doctors, the team, and patients. The procedures that occur in the emergency room are standardized, following a pattern, almost of cause and effect. While this order has the advantage of ensuring uniformity in responding to critical events, it can sometimes momentarily suppress doctors’ clinical impression. In the first approach to the present patient, an allergic reaction to tuna was considered; however, given the clinical stability and even the patient’s own demeanor, in the face of the surrounding scenario, we questioned the diagnosis. This doubt was eventually dispelled by the mother’s presentation with similar symptoms, even before the results of medication and laboratory tests were available. In emergency medicine, it is important to always assume the most serious entity; however, obtaining a carefully collected history can avoid the unnecessary expenditure of resources and patient anxiety. This case can also help doctors realize that sometimes symptoms, such as the exuberant rash in this patient (often the most valued aspect), must be framed within the context of other vital signs to define their severity.
